# The roles of plant proteases and protease inhibitors in drought response: a review

**DOI:** 10.3389/fpls.2023.1165845

**Published:** 2023-04-18

**Authors:** Sellwane Jeanette Moloi, Rudo Ngara

**Affiliations:** Department of Plant Sciences, University of the Free Sate, Qwaqwa Campus, Phuthaditjhaba, South Africa

**Keywords:** plant proteases, protease inhibitors, proteolysis, protein degradation, drought stress, drought response, comparative proteomics, protein homeostasis

## Abstract

Upon exposure to drought, plants undergo complex signal transduction events with concomitant changes in the expression of genes, proteins and metabolites. For example, proteomics studies continue to identify multitudes of drought-responsive proteins with diverse roles in drought adaptation. Among these are protein degradation processes that activate enzymes and signalling peptides, recycle nitrogen sources, and maintain protein turnover and homeostasis under stressful environments. Here, we review the differential expression and functional activities of plant protease and protease inhibitor proteins under drought stress, mainly focusing on comparative studies involving genotypes of contrasting drought phenotypes. We further explore studies of transgenic plants either overexpressing or repressing proteases or their inhibitors under drought conditions and discuss the potential roles of these transgenes in drought response. Overall, the review highlights the integral role of protein degradation during plant survival under water deficits, irrespective of the genotypes’ level of drought resilience. However, drought-sensitive genotypes exhibit higher proteolytic activities, while drought-tolerant genotypes tend to protect proteins from degradation by expressing more protease inhibitors. In addition, transgenic plant biology studies implicate proteases and protease inhibitors in various other physiological functions under drought stress. These include the regulation of stomatal closure, maintenance of relative water content, phytohormonal signalling systems including abscisic acid (ABA) signalling, and the induction of ABA-related stress genes, all of which are essential for maintaining cellular homeostasis under water deficits. Therefore, more validation studies are required to explore the various functions of proteases and their inhibitors under water limitation and their contributions towards drought adaptation.

## 1 Introduction

Plants require an optimum supply of light, water, temperature, and mineral nutrients for normal growth and development (Taiz and Zeiger, 2012). In nature, however, plants are often exposed to diverse abiotic stresses, including drought, high salinity, extreme temperatures, chemical toxicity and nutrient deficiency, which negatively affect their survival (Wang et al., 2003; Mahajan and Tuteja, 2005). In the case of crops, these stress factors may reduce yield, causing negative impacts on food supply chains (Mittler, 2006). Among the environmental stresses, drought is the major limiting factor of crop production worldwide (Farooq et al., 2009; da Silva et al., 2013), and its effects are further exacerbated by climate change and global warming. As climate models continue to predict the occurrence of frequent and severe drought episodes, more famines are likely to be experienced in drought-prone areas (IPCC, 2007; Nelson et al., 2009).

Plants are exposed to water deficit stress when rainfall declines during the growing season and when the rate of transpiration exceeds that of water absorption by roots due to hot and dry conditions (Turner and Begg, 1981; Bray, 1997). Consequently, osmotic stress develops, causing adverse effects on plant physiology, metabolism, and growth patterns (Taiz and Zeiger, 2012). For example, water deficit disrupts cell structure and function, including membrane integrity, photosynthesis, respiration, and growth processes (Anjum et al., 2011; Kumar et al., 2018; Kapoor et al., 2020). The extent of such effects, however, depends on the duration and severity of the water limitation, the plant species, genotype and/or developmental stage (Mullet and Whitsitt, 1996; Bray, 1997; Anjum et al., 2011), and whether water scarcity occurs in combination with other biotic and/or abiotic stresses (Rizhsky et al., 2004; Suzuki et al., 2014; Zandalinas et al., 2018). Nonetheless, plants have developed various mechanisms to cope with the prevailing environmental stresses to maintain cell structure, function and growth (Xiong and Zhu, 2002; Farooq et al., 2009; Shao et al., 2009; Osakabe et al., 2014).

One of the earliest responses of plants to dehydration stress is stomatal closure which is induced by the phytohormone abscisic acid (ABA) (Zhang et al., 2006; Lim et al., 2015; Kuromori et al., 2018). ABA is synthesised in roots and leaves in response to reduced water content in drying soil (Davies and Zhang, 1991; Taiz and Zeiger, 2012). As stomata close, transpiration water loss is reduced, and water is conserved. However, stomatal closure also restricts the absorption of CO_2_ required for photosynthesis and the uptake of nutrients for plant growth (Basu et al., 2016). The reduction in CO_2_ absorption and assimilation leads to an electron-rich environment in cells that is conducive for increased accumulation of reactive oxygen species (ROS) (Salehi-Lisar and Bakhshayeshan-Agdam, 2016), causing oxidative stress (Gill and Tuteja, 2010; Sharma et al., 2012), a secondary effect of many environmental stresses, including drought (Levitt, 1980a; Levitt, 1980b). If ROS molecules are not maintained at relatively low levels and/or effectively detoxified, they may damage lipids, nucleic acids and proteins, causing catastrophic disruptions to cell structure and metabolism (Wang et al., 2003).

To mitigate the adverse effects of both osmotic and oxidative stresses on cells, plants accumulate or activate a variety of stress-related genes, proteins, and metabolites through changes in cellular metabolism (Xiong and Zhu, 2002; Shao et al., 2009). Some of these molecular changes are mediated by ABA *via* the ABA-dependent pathway of stress response, while others form part of the ABA-independent pathway (Shinozaki and Yamaguchi-Shinozaki, 1997; Shinozaki and Yamaguchi-Shinozaki, 2007; Yoshida et al., 2014), and have various signalling, gene regulatory and protective functions (Shinozaki and Yamaguchi-Shinozaki, 1997; Shinozaki and Yamaguchi-Shinozaki, 2007). Ultimately, cellular homeostasis is maintained to promote plant survival under stressful conditions (Xiong and Zhu, 2002; Vinocur and Altman, 2005).

Apart from ABA, other phytohormones such as jasmonic acid, salicylic acid, ethylene, auxins, gibberellins, cytokinins, brassinosteroids, and small molecular peptides also mediate plant responses to drought through complex interactions of their signalling pathways (Clarke and Durley, 1981; Hale and Orcutt, 1987; Ullah et al., 2018; Jogawat et al., 2021; Salvi et al., 2021; Iqbal et al., 2022). The crosstalk between phytohormones may have positive or negative effects on the interacting hormones and their ultimate effect in alleviating drought stress (Ullah et al., 2018; Jogawat et al., 2021). Furthermore, the biosynthesis, catabolism, and transport of phytohormones, and their conversion between bioactive, inactive, and storage forms also influence how plants reprogram growth and developmental processes to survive drought stress (Clarke and Durley, 1981; Hale and Orcutt, 1987). For example, the levels of ABA, auxins, salicylic acid, jasmonic acid, brassinosteroids, and ethylene may increase in response to water deficits to facilitate stomata regulation of transpiration, osmotic adjustment, ROS scavenging, and increased root growth.

Conversely, the contents of gibberellins and cytokinins tend to decline under drought, and these hormones have opposite effects on stomata conductance and shoot and root meristem activity compared to ABA (Clarke and Durley, 1981; Hale and Orcutt, 1987; Ullah et al., 2018; Salvi et al., 2021; Iqbal et al., 2022). Furthermore, a decline in gibberellins content in plants under drought results in the accumulation of growth-repressor proteins such as DELLA and the subsequent development of growth-retarded plant phenotypes that are more tolerant to drought (Achard and Genschik, 2009; Salvi et al., 2021). Likewise, drought-induced reduction in cytokinins is associated with shoot growth inhibition and enhanced root growth facilitating water absorption from drying soils (Salvi et al., 2021). Collectively, phytohormone interactions in plants under drought stress modulate plant responses through complex morpho-physiological and molecular mechanisms, including transcriptional regulation of drought stress-related genes to maximise survival.

Research on plant responses to drought has increased exponentially in the last two decades, as evidenced by the growing number of related publications available on the PubMed database https://pubmed.ncbi.nlm.nih.gov/. In addition, genomes of over 788 different plant species (Sun et al., 2022), including those of the model plant Arabidopsis (*Arabidopsis thaliana*) (The Arabidopsis Genome Initiative, 2000), and major crops (Bolger et al., 2014) have been sequenced. Several reviews have discussed the applications of genome sequences (Edwards and Batley, 2010; Bolger et al., 2014) and genome editing tools (Arora and Narula, 2017; Bao et al., 2019; Zhang et al., 2020) for crop improvement, including drought resilience. Furthermore, genome sequence data are invaluable reference tools for high-throughput “omics” studies involving many aspects of plant biology. Consequently, innumerable transcriptomics, proteomics, and metabolomics studies on plant responses to drought have been published and reviewed (Cramer et al., 2011; Ngara and Ndimba, 2014; Shanker et al., 2014; Barkla, 2016; Ngara et al., 2021; Singh et al., 2022; Thanmalagan et al., 2022). In addition, the generalised functional catalogue of plant genes outlined by Bevan et al. (1998) has become an invaluable tool for assigning putative roles to drought-responsive proteins identified in proteomics studies (Nouri and Komatsu, 2010; Aranjuelo et al., 2011; Zhao et al., 2011; Mohammadi et al., 2012a; Wang et al., 2014; Tamburino et al., 2017; Goche et al., 2020).

Undoubtedly, the drought models employed in such studies are quite diverse, and so are the results generated (Osmolovskaya et al., 2018). Nonetheless, “omics” studies continue to broaden our understanding of how plants reprogram cellular metabolism to maximise survival under unfavourable environmental conditions (Kido et al., 2016; Ngara et al., 2021; Baldoni, 2022; Rakkammal et al., 2022; Singh et al., 2022). Furthermore, comparative proteomics studies of plant genotypes with contrasting drought phenotypes provide new insights into drought response mechanisms (Barkla, 2016). For example, common plant responses to water deprivation between drought-tolerant and sensitive cultivars include the down-regulation of metabolism-related proteins, possibly as an energy-saving mechanism, and the up-regulation of defence-related proteins for protective functions (Ford et al., 2011; Jedmowski et al., 2014; Faghani et al., 2015; Cheng et al., 2016; Wu et al., 2016; Zeng et al., 2019; Goche et al., 2020; Moosavi et al., 2020). Conversely, increased alternative splicing events (Fracasso et al., 2016) and higher constitutive expression of secondary metabolism, redox homeostasis, and translation-related genes (Fracasso et al., 2016; Azzouz-Olden et al., 2020) possibly contribute towards the drought-superior traits of some varieties. These tolerant varieties also exhibit greater drought-induced accumulation of proteins involved in signal transduction, osmolyte biosynthesis, transcription, translation, and several protective roles such as antioxidant enzymes, dehydrins, late embryogenesis abundant proteins, chaperons, and regulators of proteolysis (Wang et al., 2015; Cheng et al., 2016; Chmielewska et al., 2016; Goche et al., 2020). Overall, such genes and proteins are pivotal during drought adaptation (Ingram and Bartels, 1996; Ramanjulu and Bartels, 2002) and could serve as potential biomarkers for crop improvement strategies (Barkla, 2016).

Here, we review the differential expression and functional activities of plant protease and protease inhibitor proteins under drought stress, mainly focusing on comparative studies of cereal crops involving genotypes of contrasting drought phenotypes. We further explore studies of transgenic plants that either overexpress or repress proteases or their inhibitors under drought conditions and discuss the potential roles of these transgenes in drought response.

## 2 Plant proteases and protease inhibitors

Plant proteases are proteolytic enzymes that hydrolyse peptide bonds in proteins and are found in various plant tissues and organs (Palma et al., 2002; Schaller, 2004; van der Hoorn, 2008; Sharma and Gayen, 2021). Their activities are tightly regulated through the transcriptional control of protease transcripts, post-translational modifications of their proenzymes, actions of endogenous protease inhibitors, and/or compartmentalization into organelles and cellular compartments to avoid random acts of protein degradation (Brzin and Kidrič, 1996; Vierstra, 1996; Diaz-Mendoza et al., 2016). For instance, plant proteases are located in the cytosol, chloroplasts, vacuoles, nuclei, endoplasmic reticulum, proteasome, mitochondria, and cell walls (Vierstra, 1996; Kidric et al., 2014; Diaz-Mendoza et al., 2016), and also secreted into the extracellular matrix (Ngara et al., 2018; Ngcala et al., 2020; Godson and van der Hoorn, 2021). Each of these cellular compartments may possess specialised proteolytic pathways. For example, in the cytosol, protein degradation is mainly carried out by the highly selective ubiquitin-proteasome system (UPS), which consists of ubiquitin, the proteasome and associated components (Hopkins and Huner, 2009; Xu and Xue, 2019). Other proteolytic machinery found in plants include the caseinolytic protease (Clp) system in plastids and mitochondria and the vacuolar processing enzymes in vacuoles (Kato and Sakamoto, 2010; Vaseva et al., 2012; Nishimura and van Wijk, 2015; van Wijk, 2015; Ali and Baek, 2020).

Proteases are structurally and functionally diverse and are classified based on their catalytic activity, such as aspartic, cysteine, serine and threonine peptidases (Callis, 1995; Schaller, 2004). Alternatively, proteolytic enzymes are grouped into endo- and exo-peptidases depending on the site of cleavage on the peptide chain (Palma et al., 2002; Schaller, 2004). Examples of endopeptidases include serine, cysteine, aspartic, threonine and metalloendopeptidases, while exopeptidases include aminopeptidases, dipeptidases, carboxypeptidase, didpeptidyl-peptidases, omega peptidases and peptidyl-dipeptidases (Beers et al., 2000; Palma et al., 2002; Vaseva et al., 2012; Kidric et al., 2014). Likewise, protease inhibitors are diverse small molecules of either protein or non-protein nature (Polya, 2003); and are located in various plant organs and cellular compartments (Mosolov and Valueva, 2011; Kidric et al., 2014). They are differentiated based on their reaction mechanism or the type of proteases they inhibit (Mosolov and Valueva, 2011; Kidric et al., 2014). Examples of endogenous plant protease inhibitors include phytocystatins, serpins, Kunitz protease inhibitors, Bowman-Birk inhibitors, a-amylase inhibitors, bifunctional trypsin inhibitors, metallocarboxypeptidase inhibitors, mustard trypsin inhibitors, and potato-type inhibitors (Mosolov and Valueva, 2011; Vaseva et al., 2012; Kidric et al., 2014; Hellinger and Gruber, 2019). Additional information and online resource tools for plant proteases and their endogenous inhibitors can be found in the listed databases (**Table 1**).

**Table 1:** Online databases and resource tools for plant proteases and protease inhibitors.

Database/resource tool	Website	Supported information	References
MEROPS	http://merops.sanger.ac.uk	Proteolytic enzymes, their substrates and inhibitors	Rawlings et al. (2018)
PLANT-PIs	http://plantpis.ba.itb.cnr.it/	Proteases inhibitors and their genes in higher plants	De Leo et al. (2002)
PINIR	http://pinir.ncl.res.in	Potato type inhibitor-II proteins	Yadav et al. (2021)
ProtIdent	http://www.csbio.sjtu.edu.cn/bioinf/Protease/	Proteolytic enzymes	Chou and Shen (2008)

### 2.1 Putative protein substrates and functions of plant proteases

The mechanism of action of proteases, their inhibitors, and different proteolytic pathways in plants have been extensively reviewed elsewhere (Vierstra, 1996; Vaseva et al., 2012; Kidric et al., 2014) and can be accessed for in-depth reading. Other reviews have discussed proteolytic machinery in plastids, mitochondria and peroxisomes, as well as their roles in maintaining protein homeostasis, also called proteostasis in these organelles (Kato and Sakamoto, 2010; Nishimura and van Wijk, 2015; van Wijk, 2015; Nishimura et al., 2016; Nishimura et al., 2017; Sun et al., 2021). The extensive review by van Wijk (2015) also lists substrates of plastidal, mitochondrial and peroxisomal proteases and is recommended for further reading, together with other references therein. Readers are also encouraged to access the comprehensive review chapter by Kato and Sakamoto (2010) on major chloroplast proteases, their substrates and functions. Nishimura et al. (2016) also provide a comprehensive pictorial depiction of the different types of metallo, serine, and aspartic proteases in plastids of land plants, their suborganellar location, and apply case studies to illustrate how the major chloroplastic proteases maintain organellar proteostasis. These earlier reviews highlight the different types of proteolytic machinery in plant cells, organelles and compartments, as well as the integral role of proteolysis during protein processing, maturation and quantity and quality control.

A common notion from these reviews is the extensive diversity of plant proteases, their multiple protein substrates, subcellular locations, and specialised functions. These proteolytic systems also work in a coordinated manner to maintain protein homeostasis (Kato and Sakamoto, 2010). However, the identities of specific protein substrates and mechanisms of action for many of these proteases are only partially known (Kato and Sakamoto, 2010; van Wijk, 2015). Nevertheless, enormous progress has been made in profiling the physiological substrates of various plant proteases. For example, several studies have used *in vitro* recombinant proteins, mutant systems, substrate-trapping assays, and N-terminal degradome techniques coupled with quantitative proteomics and mass spectrometry technologies for substrate identification (Moberg et al., 2003; Tsiatsiani et al., 2013; Sako et al., 2014; Carrie et al., 2015; Galiullina et al., 2015; Opalinska et al., 2017; Moreno et al., 2018; Welsch et al., 2018; Rowland et al., 2022).

In a recent study, Rowland et al. (2022) used several mutant types, the terminal amine stable isotopic labelling of substrates (TAILS), and label-free proteomic methods to investigate the coordination between Clp and presequence protease (PreP) proteases in maintaining chloroplast proteostasis. The results showed synergistic interactions between the two protease systems, their effects on embryo lethality, and changes in N-terminal protein processing activities. Furthermore, the loss-of-function of the Clp systems resulted in the over-accumulation of chloroplast stromal chaperons such as heat shock protein 90 (HSP90), chaperon protein CLPB3, chloroplastic (CLPB3) and chaperonins (CPN60/10/20), which possibly re-fold and stabilise abnormally folded and aggregated proteins. However, none of these three chaperons was affected in the *prep1prep2* mutant plants relative to the wild-type (Rowland et al., 2022) further highlighting the different effects of proteases in plants. Using several *clp* mutants, co-immunoprecipitation, and proteomic analyses, Welsch et al. (2018) experimentally showed that phytoene synthase, an enzyme in carotenoid biosynthesis, is a substrate for the Clp protease system. Therefore, the authors argued that proteolysis is critical in ensuring quantity control of phytoene synthase for adequate carotenoid biosynthesis (Welsch et al., 2018).

We acknowledge that a complete listing of known substrates of various plant proteases is beyond the scope of the current review. However, below is a brief account of how plants maintain protein homeostasis using diverse proteolytic machinery and their substrates. In many cases, they operate in tandem to complete target protein degradation (Kato and Sakamoto, 2010; van Wijk, 2015). A summary list of protein substrates of selected plant proteolytic machinery is also presented in **Table 2**. For example, once imported into their target organelles, nucleus-encoded chloroplast and mitochondrial proteins are further processed through the proteolytic cleavage of chloroplast transit peptides (cTP) and mitochondrial targeting peptides (mTP) before maturation. These activities are carried out by the stromal processing peptidase (SPP) (Richter and Lamppa, 1999; Richter et al., 2005) and mitochondrial processing peptidase (MPP) (Ghifari et al., 2019) metalloproteases in chloroplasts and mitochondria, respectively or other yet to be identified peptidases (Rowland et al., 2022). In peroxisomes, the cleavable N-terminal peroxisomal targeting signal (PTS2) of nucleus-encoded proteins is cleaved off by the degradation of the periplasmic protein (Deg)15 proteases, while PTS1 in non-cleavable (Schuhmann et al., 2008). Deg proteases are ATP-independent serine endopeptidases with essential roles in protein quality control in nearly all organisms, including plants (Schuhmann et al., 2008; Schuhmann et al., 2012).

**Table 2:** Examples of protein substrates for selected proteases and peptidases during plant growth and development.

Name	Class	Examples of substrates^1^	Physiological functions of proteolytic activity	References^2^
**Clp**	ATP-dependent serine-type protease	Phytoene synthase; molecular chaperons, CAO, various chloroplast proteins involved in protein homeostasis, photosynthesis, redox homeostasis, plastid biosynthesis pathway, and plastid gene expression	Chloroplast biogenesis, chloroplast housekeeping, protein homeostasis, regulation of chlorophyll b synthesis, protein quality control, folding, and activity, quantity control for adequate carotenoid biosynthesis	Kato and Sakamoto (2010); van Wijk, 2015; Moreno et al. (2018); Welsch et al. (2018); Liao and van Wijk (2019); Rodriguez-concepcion et al. (2019); Bouchnak and van Wijk (2021); Rowland et al. (2022)
**Deg**	ATP-independent serine-type protease	Thylakoid lumen proteins, plastocyanin, OEC33 of PSII; photo-damaged, heat and high light intensity pretreated D1 protein of the PSII	PSII repair cycle, response to photo-oxidative stress, maintenance of chloroplast homeostasis	Kato and Sakamoto (2010)
**FtsH**	ATP-dependent metalloprotease	D1 protein of PSII; oxidatively damaged membrane proteins, MPC4, Pam18-2, Tim17-2, oxidatively damaged mitochondrial proteins, Slp1& 2, GCD, several metabolism-related proteins	PSII repair cycle, plastid development and thylakoid membrane formation, Response to photo-oxidative stress, high light acclimation, senescence, thermotolerance	Kato and Sakamoto (2010); Opalinska et al. (2017)
**Metacaspase9**	Cysteine aspartate protease	Several substrates including PEPCK, LEAs, chaperons, proteases, protease inhibitors, HSPs, Toc159, protein synthesis-related proteins, metacaspase	PCD, maintenance of plant growth and development, regulation of cell metabolism, possible involvement in drought stress response	Tsiatsiani et al. (2013)
**Icp55**	N-terminal aminopeptidase	Several substrates including HSPs; antioxidant enzymes, proteases, PRR proteins, ATP synthases, metabolism and protein synthesis-related proteins	Secondary processing of mitochondrial presequences; maintenance of protein stability in mitochondria	Carrie et al. (2015)
**Oct1 (or MIP)**	N-terminal aminopeptidase	PRR proteins, valyl-tRNA synthetase, PRORP1, PMH1, PMH2, B13 NADH complex, glycine-tRNA synthetase	Secondary processing of mitochondrial presequences; maintenance of protein stability in mitochondria	Carrie et al. (2015)
**Phytaspase**	Subtilisin-like serine-dependent protease	Various peptide-based caspase substrates	PCD triggered by biotic and abiotic factors such oxidative and salt stresses	Galiullina et al. (2015)
**Plsp1**	Serine endopeptidase	Toc75, OEC33	Biogenesis of chloroplast internal membranes, protein maturation	Kato and Sakamoto (2010)
**PreP**	Metalloendopeptidase	Cleaved cTP, mTP and PTS2 peptides	Prevention of over-accumulation of potentially toxic non-functional peptides; amino acid recycling	Moberg et al. (2003); Kato and Sakamoto (2010); Kmiec and Glaser (2012); van Wijk (2015); Rowland et al. (2022)
**26S proteasome**	ATP-dependent protease complex	DELLA, JAZ, LTA2, PDH E1α; various unimported or misfolded plastid precursor proteins in the cytosol	Regulation of phytohormone activity and signaling. Degradation of unimported and misfolded plastid precursors to avoid cytotoxicity, maintenance of cellular homeostasis	Achard and Genschik (2009); Sako et al. (2014)
**VPE**	Cysteine-type endopeptidase	Proprotein precursors of various vacuolar proteins such as seed storage proteins, hydrolytic enzymes, protease inhibitors, stress proteins e.g. chitinases	Maturation and activation of various vacuolar proteins, mediation of PCD, production of cyclic peptides due to peptide ligating activity, supports plant development and responses to environmental stresses, processing of seed storage proteins, and hydrolytic enzymes, protease inhibitors	Yamada et al. (2020)

^1^To manage long lists of protein substrates from the reviewed research paper, major functional groups of substrates are given; ^2^The list of protein substrates was retrieved from both research papers and reviews.

Caspases-cysteine-dependent death and inflammation-related proteases of animal origin; CAO, chlorophyll a oxidase; Clp, caseinolytic protease; cTP, chloroplast transit peptide; Deg, degradation of periplasmic proteins; FtsH, filamentous temperature-sensitive H; HSPs, heat shock proteins; Icp55, intermediate cleaving peptidase of 55 kDa; JAZ, jasmonate ZIM-domain; LEA, late embryogenesis abundant; LTA2, nuclear encoded dihydrolipoamide s-acetyltransferase encoding the pyruvate decarboxylase E2 subunit; MIP, mitochondrial intermediate peptidase; MPC4, mitochondrial pyruvate carrier 4; mTP, mitochondrial targeting peptides; Oct1, octapeptidyl aminopeptidase 1; OEC33, oxygen-evolving complex protein 33; PAM18-2, presequence translocase-associated motor 18-2; PCD, programmed cell death; PDH E1α, pyruvate dehydrogenase E1 alpha subunit; PlsP1, plastidal type-I signal peptidase; PMH1, mitochondrial RNA helicase 1; PMH2, mitochondrial RNA helicase 2; PreP, presequence protease; PRORP1, proteinaceous RNase P1; PRR, pentatricopeptide repeat; PSII, photosystem II; PTS2, N-terminus peroxisomal targeting signal; Slp1, stomatin-like protein 1; Slp2, stomatin-like protein 2; TIM-17-2, translocase inner membrane subunit 17-2; Toc75, translocon at the outer envelope membrane of chloroplast 75; Toc159, translocon at the outer envelope membrane of chloroplast 159; VPE, vacuolar processing enzyme.

Schuhmann et al. (2008) experimentally verified that PTS2-containing presequences of glyoxysomal malate dehydrogenase, 3-keto-acyl-CoA thiolase, and a long-chain acyl-CoA synthetase 6 are substrates of Deg15. The cleaved cTP, mTP and PTS2 peptides are subsequently degraded by PreP (Moberg et al., 2003; Kmiec and Glaser, 2012) or organellar oligopeptidase (OPP) (Ghifari et al., 2019) to prevent the accumulation of potentially toxic non-functional peptides and to facilitate amino acid recycling (van Wijk, 2015; Rowland et al., 2022). Other *in vitro* studies using recombinant proteins have shown that various Deg family proteins may be involved in maintaining chloroplast proteostasis by degrading thylakoid lumen proteins such as plastocyanin and the oxygen-evolving complex (OEC) protein 33 of the photosystem II (PSII), and heat stressed or photo-damaged D1 reaction centre protein (Kato and Sakamoto, 2010; van Wijk, 2015). Chloroplasts and mitochondria also contain other specialised endopeptidases, such as the type-I signal peptidase family (SPase I) and the plastidal type-I signal peptidase (Plsp1), that process luminal and intermembrane precursor proteins by cleaving off signal peptides for full maturation and activation (Tuteja, 2005; Kato and Sakamoto, 2010). For example, Plsp1 is involved in the maturation of the translocon at the outer envelope membrane of chloroplasts, 75kDA (Toc75), a member of the protein translocation complex at the outer envelope membrane of plastids (Baldwin et al., 2005). Other chloroplast and mitochondrion-encoded proteins are further post-translationally modified by the proteolytic removal of an N-terminal methionine using methionine aminopeptidases (MAPs) to achieve normal protein function and leaf development (Ghifari et al., 2019).

**Figure 1:**
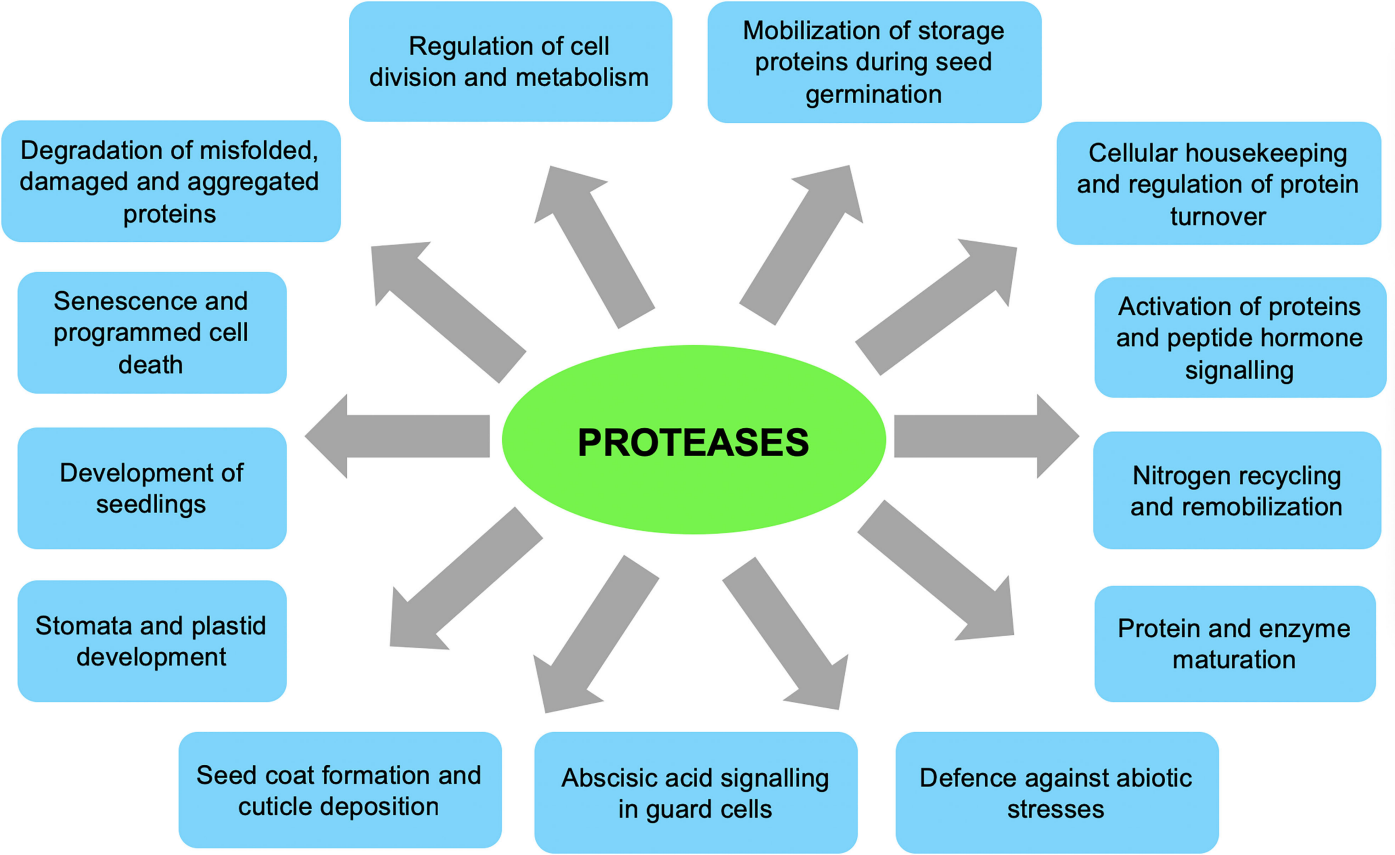
General functions of plant proteases in plant growth and development. (Brzin and Kidrič, 1996; Vierstra, 1996; Schaller, 2004; van der Hoorn, 2008; Hopkins and Huner, 2009; Kato and Sakamoto, 2010; Kidric et al., 2014; van Wijk, 2015; Diaz-Mendoza et al., 2016; Sharma and Gayen, 2021).

Due to their wide structural diversity, substrate specificities, selectivity, and/or subcellular locations (Vierstra, 1996; Kato and Sakamoto, 2010; Vaseva et al., 2012; Kidric et al., 2014; van Wijk, 2015), plant proteases are involved in numerous functions during normal plant growth and development and in response to biotic and abiotic stresses (**Figure 1**). For instance, proteolytic activities are essential during nitrogen recycling and remobilisation, leaf senescence and programmed cell death, controlled degradation of damaged or abnormal proteins, activation and maturation of proteins and peptide hormones, seed germination, seedling growth, cellular housekeeping, regulating protein turnover, and in response to biotic and abiotic stresses (Brzin and Kidrič, 1996; Vierstra, 1996; Schaller, 2004; van der Hoorn, 2008; Hopkins and Huner, 2009; Kato and Sakamoto, 2010; Kidric et al., 2014; van Wijk, 2015; Diaz-Mendoza et al., 2016). On the other hand, protease inhibitors regulate the function of proteases by inhibiting their catalytic activity and may participate in plant defence and protective roles in response to biotic and abiotic stresses (Brzin and Kidrič, 1996; Mosolov and Valueva, 2005; Mosolov and Valueva, 2011).

### 2.2 Plant protease and protease inhibitor proteins under drought stress

#### 2.2.1 Comparative proteomics studies

The involvement of plant proteases and protease inhibitors in drought response has been reported in various plant species using transcriptomics, proteomics and/or enzyme activity assays. Earlier reviews have also discussed the roles of plant proteases and their inhibitors under normal growth and in response to various biotic and abiotic stresses (Brzin and Kidrič, 1996; Mosolov and Valueva, 2011; Kidric et al., 2014; Godson and van der Hoorn, 2021), including senescence (Diaz-Mendoza et al., 2016). Xu and Xue (2019) reviewed the components of the UPS and their regulation in response to biotic and abiotic stresses. Others have outlined the potential function of plant proteases in signal transduction systems under environmental stresses (Sharma and Gayen, 2021), including drought (D’Ippólito et al., 2021). Due to the extensive collection of “omics” studies available in the public domain, we opted to focus this review on the drought-induced expressional changes of protease and protease inhibitor proteins as depicted in comparative proteomic studies of cereal crops (**Table 3**). However, the general literature on proteomics approaches (Monteoliva and Albar, 2004; Wu et al., 2006; Abdallah et al., 2012; Zargar et al., 2016) and plant proteome analyses under drought stress and applications in crop improvement (Mohammadi et al., 2012b; Barkla, 2016; Wang et al., 2016; Ghatak et al., 2017) has been extensively reviewed elsewhere and will not be discussed in the current review.

**Table 3:** Studies showing differential accumulation and functional activities of protease and protease inhibitor proteins in plants under drought stress.

Plant species	Tissue	Genotypes	Proteomic methods	Differential expression of proteases and inhibitor proteins under drought stress	References
Sorghum					
	Roots	SA1441-tolerant ICSB338-suseptible	iTRAQ, LC-MS/MS, qRT-PCR	Up-regulation of peptidase C1A family in both varieties, aspartic peptidase A1 family peptidase in the susceptible variety, and proteinase inhibitor, potato inhibitor I in the tolerant variety.	Goche et al. (2020)
	Leaves	11434-tolerant 11431-sensitive	2D-DIGE, MALDI-TOF-MS	Up-regulation of aspartate protease, mitochondrial processing peptidase in the susceptible variety.	Jedmowski et al. (2014)
**Rice**	Leaves	IAC1131-tolerant Nipponbare-sensitive	SDS PAGE, Label-free, TMT and western blotting analyses	Up-regulation of ClpD1 protease in the drought tolerant variety.	Wu et al. (2016)
Wheat					
	Leaves	Xihan No.2-tolerant Longchun 23- sensitive	2D gel electrophoresis, MALDI-TOF/TOF MS	Up-regulation of proteasome subunit alpha type-7-A, proteasome subunit alpha type-1 and ATP-dependent Clp protease proteolytic subunit in the drought sensitive variety. The drought tolerant variety increased the abundance of proteasome subunit alpha type-2, 20S proteasome beta 7 subunit, aspartic proteinase nepenthesin-1 precursor and triticain alpha subunit.	Cheng et al. (2016)
	Leaves Roots	SERI M 82-tolerant SW89.5193-sensitive	2D gel electrophoresis, MALDI-TOF/TOF MS, qRT-PCR	Up regulation of proteasome alpha type protein in leaves of the drought sensitive variety.	Faghani et al. (2015)
	Leaves	Excalibur-tolerant RAC875-tolerant Kukri-sensitive	iTRAQ and LC-MS/MS	Up-regulation of type II metacaspase and leucine aminopeptidase in drought-tolerant cultivars.	Ford et al. (2011)
Barley					
	Leaves	15141-tolerant 15163-sensitive	2D gel electrophoresis, MALDI-TOF/TOF MS	Up-regulation of ATP-dependent Clp proteases and leucine aminopeptidase in both varieties, while zinc metalloprotease increased in abundance in the drought tolerant variety.	Ashoub et al. (2013)
	Leaves	XZ5-tolerant XZ54-sensitive	2D gel electrophoresis, MALDI-TOF-TOF, qRT-PCR	Up-regulation of FtsH protease 1 metallopeptidase in the drought tolerant genotype.	Wang et al. (2015)
	Leaves Roots	Cam/B1/CI-tolerant Maresi-sensitive	2D gel electrophoresis, MALDI-TOF-TOF	Up-regulation of ClpP protease in the leaves of the drought tolerant variety.	Chmielewska et al. (2016)

SDS-PAGE, sodium dodecyl sulphate-polyacrylamide gel electrophoresis; TMT, Tandem Mass Tags; iTRAQ, isobaric tags for relative and absolute quantification; LC-MS/MS, Liquid chromatography mass spectrometry; 2D-DIGE, two- dimensional difference gel electrophoresis qRT-PCR-quantitative real time polymerase chain reaction; MALDI-TOF-MS, matrix assisted laser desorption ionization-time of flight mass spectrometry; Clp, caseiolytic protease; FtsH, filamentous temperature sensitive H; ATP, adenosine triphosphate.

Wu et al. (2016) used label-free and tandem mass tag multiplexing methods to analyse leaf proteome changes in two rice (*Oryza sativa*) varieties subjected to water deficits and re-watering. Amongst the observed unique proteome changes was the increased accumulation of a ClpD1 protease in the drought-tolerant IAC1131 rice variety under severe drought conditions and its decreased accumulation upon re-watering. ClpD1 belongs to the caseinolytic protease (Clps) family of proteins (Kato and Sakamoto, 2010; Roberts et al., 2012; Vaseva et al., 2012; Nishimura and van Wijk, 2015) and is encoded by the drought-inducible gene *OsClpD1* in rice (Wu et al., 2016). Clp proteases are involved in degrading damaged proteins in the plastids (Kato and Sakamoto, 2010; Ali and Baek, 2020). Wu et al. (2016) suggested that the drought-induced accumulation of ClpD1 in the drought-tolerant variety increased the genotype’s chances of coping with extreme drought conditions through the regulation of protein quality.

In a comparative gel-based proteomic study, an array of proteolysis-related proteins were up-regulated in leaves of wheat (*Triticum aestivum*) plants subjected to drought stress for 18, 24 and 48 hours (Cheng et al., 2016). For example, proteasome subunit alpha type-2, 20S proteasome beta 7 subunit, an aspartic proteinase precursor protein and a triticain alpha subunit were up-regulated in Xihan No. 2, a drought-tolerant wheat variety. Likewise, increased abundances of proteasome subunit alpha type-7-A, proteasome subunit alpha type-1 and an ATP-dependent Clp protease proteolytic subunit were observed in the drought-sensitive variety following water deprivation. The authors suggested that proteolysis is an essential mechanism for maintaining protein quality under stress, irrespective of a cultivar’s degree of drought resilience. Furthermore, when coupled with adequate protein refolding by chaperons, proteolytic activity helps plants to maintain protein homeostasis under dehydration stress (Cheng et al., 2016). In another study, a proteasome alpha-type protein accumulated in leaves of a drought-sensitive wheat variety but not the drought-tolerant one upon exposure to water limitation (Faghani et al., 2015). Overall, the observations highlight the importance of selective protein degradation by the ubiquitin-proteasome system in plants under water deficit stress.

In another comparative gel-based proteomic study of sorghum (*Sorghum bicolor*) leaves, an aspartate protease and a mitochondrial processing peptidase were up-regulated in the drought-susceptible variety in response to drought stress (Jedmowski et al., 2014). Aspartic proteases are widely distributed within the plant kingdom and are involved in protein degradation during normal plant development, nitrogen recycling, programmed cell death, and stress response (Mutlu and Gal, 1999; Simoes and Faro, 2004). As discussed earlier, mitochondrial processing peptidases are involved in the removal of N-terminal signal peptides also called the presequence, from nuclear-encoded mitochondrial proteins during or after their import into the mitochondrion (Glaser et al., 1998; Kwasniak et al., 2012; Ghifari et al., 2019). As such, the observed drought-increased accumulation of aspartic protease could indicate high levels of degraded proteins in the susceptible variety, while the mitochondrial processing peptidase could be responsible for presequence removal (Jedmowski et al., 2014), and thus maturation of various newly synthesised mitochondrial precursors (Glaser et al., 1998; Gakh et al., 2002; van Wijk, 2015).

In one of our studies, Goche et al. (2020) conducted a comparative root proteomic analysis of two contrasting sorghum varieties subjected to limited watering. Among the observed differences in protein expression patterns were the up-regulation of proteolytic enzymes in ICSB338, the drought-sensitive variety, while the more drought-tolerant SA1441 accumulated proteins involved in transcription, protein synthesis, and the inhibition of protease activities (Goche, 2018; Goche et al., 2020). For example, dipeptidyl peptidase, serine-type, cysteine-type, and aspartic-type endopeptidases were up-regulated in the drought-susceptible sorghum variety upon exposure to drought stress. On the other hand, the drought-tolerant variety predominantly up-regulated protease inhibitors such as the proteinase inhibitor, potato inhibitor I (Goche, 2018; Goche et al., 2020). Although the significance of such contrasting proteome expression patterns warrants further functional validation studies, it is evident that the drought-resilient SA1441 reprograms gene expression, possibly to facilitate the accumulation of diverse stress proteins and protect them from proteolytic degradation. It is also plausible that the drought-sensitive sorghum variety requires extensive protein degradation to remove damaged proteins in many cellular locations and to recycle amino acids for targeted protein synthesis (Hopkins and Huner, 2009).

Ford et al. (2011) also conducted an extensive comparative leaf proteomic analysis of one drought-sensitive Kukri and two drought-tolerant RAC875 and Excalibur wheat cultivars in response to cyclic drought treatment. Leaf samples were subsequently taken during the initial period of water deficit, two wilting time-points, and following re-watering for relative water content (RWC) estimations and proteome analysis. As observed from the RWC results, this experimental system provided a platform for investigating complex physiological changes of wheat under repetitive cycles of water deficit and recovery, similar to those experienced under natural field conditions. Comparative analyses of the drought-responsive proteins were conducted at the four sampling time points using isobaric tags for relative and absolute quantification (iTRAQ) and tandem mass spectrometry. Overall, the results revealed complex proteome responses of wheat to the cyclic drought treatment, genotypic differences between cultivars irrespective of their levels of drought resistance, and some common trends in metabolic changes under conditions of water deprivation (Ford et al., 2011). For example, proteins related to photosynthesis and the Calvin cycle were down-regulated across the three cultivars, while ROS-scavenging enzymes and dehydrins were up-regulated.

The study also revealed increased accumulation of type II metacaspase and leucine aminopeptidase proteins in drought-tolerant cultivars, especially towards the later stages of water deprivation. However, after re-watering, the type II metacaspase protein levels reverted to control levels in both drought-tolerant cultivars but increased in the drought-susceptible cultivar (Ford et al., 2011). Conversely, the accumulation pattern of the leucine aminopeptidase protein was not consistent between the tolerant cultivars during the water deficit but increased upon re-watering in both cultivars. Generally, the study highlights the complexity of plant proteome responses during drought stress and recovery, the diversity of proteases and their selective functions during drought stress, and the differential responses of proteases in drought-stressed wheat plants with different drought tolerance levels. For example, since metacaspases play a role in programmed cell death (Tsiatsiani et al., 2011), their early expression in drought-tolerant cultivars may implicate them in protein degradation processes that rapidly sacrifice some cells to ensure plant survival under stress (Ford et al., 2011). In addition, leucine aminopeptidases activate proteins and regulate protein turnover in plants (Walling and Gu, 1996; Matsui et al., 2006). As such, their increased accumulation upon re-watering in the drought-tolerant cultivars may indicate the importance of protein degradation, amino acid recycling and activation processes as cells reset their protein component during recovery from stress.

In another comparative proteomic study, proteases were differentially expressed in barley (*Hordeum vulgare*) plants subjected to drought stress (Ashoub et al., 2013). The study used gel-based proteomic methods to analyse the leaf proteome changes between accessions #15141 and #15163, which are tolerant and sensitive to drought, respectively. For example, two ATP-dependent Clp proteases were up-regulated in both varieties in response to drought stress. In contrast, a third ATP-dependent Clp protease and two leucine aminopeptidases were up-regulated only in the drought-susceptible accession, while a zinc metalloprotease had increased accumulation only in the tolerant accession. The authors also reported that the drought-susceptible variety showed an increased accumulation of proteases in drought response compared to the drought-tolerant variety, which accumulated more proteins involved in protective mechanisms against drought stress (Ashoub et al., 2013). Overall, the studies mentioned above emphasize the diversity of proteolytic enzymes, their selective specificities, and pivotal roles in various protein degradation processes during drought response in plants.

Although the current review aimed at surveying the drought-induced expression changes of protease and protease inhibitor proteins as depicted in comparative proteomic studies, we also scanned through a few similar studies on comparative transcriptomics to establish trends in transcript levels of these two groups of proteins. Indeed, the correlation between mRNA and proteins of various biological systems, including the human liver (Anderson and Seilhame, 1997), yeast (Gygi et al., 1999), and plants (Carpentier et al., 2008; Ponnala et al., 2014) has been debated for years, with poor correlation trends being attributed to various factors including the differential turnover rates of mRNA and proteins and posttranslational modifications of proteins (Abbott, 1999; Salekdeh et al., 2002; Vogel and Marcotte, 2012). Nevertheless, different “omics” technologies and the data thereof should be regarded as complementary, as each analytical technique may over and/or under-represent particular groups of biological molecules depending on their inherent characteristics (Carpentier et al., 2008). However, we found recent comparative transcriptomics studies that reported the drought-induced differential gene expression patterns of proteases and/or their inhibitors between plant genotypes with contrasting levels of drought tolerance (Zenda et al., 2019; Abdel-Ghany et al., 2020; Bhogireddy et al., 2020; Shivhare et al., 2020).

For example, Abdel-Ghany et al. (2020) conducted an extensive comparative transcriptome analysis between two drought-resistant and two drought-sensitive sorghum varieties in response to 20% polyethylene glycol (PEG)-induced osmotic stress. The results revealed differential expression of protein degradation-related transcripts between seedlings of the two groups of genotypes in response to 1 and 6 hours of osmotic stress treatment. For example, a cysteine protease gene and a senescence-associated gene were up-regulated in all four sorghum genotypes in response to the 1 hour and 6 hours of stress, respectively, highlighting the critical role of proteolysis and senescence during drought response in plants. In addition, following 1 hour of PEG treatment, a cysteine protease inhibitor and three cysteine protease genes were among the top 20 up-regulated genes in both drought-resistant sorghum varieties. However, at 6 hours, four seed storage protein-related protease inhibitors are down-regulated in the same drought-resistant varieties. Surprisingly, no protease or protease inhibitor genes were responsive to the PEG treatments at either time point in the drought-susceptible sorghum varieties. The authors suggested that these proteases and inhibitor genes possibly functioned in early-stress responses in the drought-resistant varieties (Abdel-Ghany et al., 2020).

Bhogireddy et al. (2020) also conducted a comparative leaf transcriptome analysis of two peanut (*Arachis hypogaea* L.) genotypes subjected to drought stress. The study reported a higher constitutive expression of several drought-responsive genes including protease inhibitors and senescence-related proteases in the drought-tolerant genotype than the sensitive one. Following water deprivation, both genotypes had increased expression of several proteolysis-related genes, but the change was generally much greater in the drought-susceptible variety. The authors suggested that high constitutive expression of drought-stress genes contributes towards the increased drought resilience of the drought-tolerant peanut genotype (Bhogireddy et al., 2020), and these findings are consistent with those reported in other drought transcriptomics studies of sorghum (reviewed in Ngara et al., 2021).

Protein degradation-related genes were also differentially expressed in maize (*Zea mays*) plants subjected to limited watering (Zenda et al., 2019). For example, several UPS-related genes were up-regulated in leaves of the drought-tolerant maize genotype following stress. Furthermore, a comparative analysis of transcripts between the drought-stressed samples of the susceptible genotype versus the tolerant genotype revealed an up-regulation of several ubiquitin-related and the really interesting new gene (RING) zinc-finger protein related genes. The authors suggested that protein ubiquitination and proteolytic processes are critical in facilitating protein turnover and homeostasis in plants under drought stress (Zenda et al., 2019). While the above account describes the trends in a few studies, future systematic reviews with meta-analyses of various studies must evaluate and synthesise the trends in constitutive and drought-induced expression of these proteolysis-related genes and proteins between multiple plants and genotypes.

#### 2.2.2 Functional validation studies

Indeed, proteomics technologies continue to broaden our understanding of plant molecular changes under drought. Such alterations affect many functional classes of plant genes (Bevan et al., 1998), including protein degradation. However, expression proteomics data still needs to be validated to ascertain the biological functions of the identified stress-responsive proteins (Rabilloud and Lescuyer, 2014; Handler et al., 2018). Undoubtedly, such proteomic to biological inferences are laden with hurdles that have been critically reviewed by Rabilloud and Lescuyer (2014). Nevertheless, different system biology approaches (Kitano, 2002), such as western blotting, quantitative polymerase chain reaction (qPCR), and enzyme activity assays, are often used to validate quantitative proteomics data (Rabilloud and Lescuyer, 2014; Handler et al., 2018). Other functional validation approaches include transgenic plant systems that either overexpress or repress specific genes under altered environmental conditions (Oh et al., 2005; Kondou et al., 2010; Bolle et al., 2011; Rhee and Mutwil, 2014; Watanabe and Hoefgen, 2019). Therefore, here we discuss a few studies that have utilised protease activity assays in cereals and legumes (**Table 4**), and transgenic overexpression (**Table 5**) and knockout or knockdown mutant (**Table 6**) plant systems to elucidate the involvement and functions of proteases and/or protease inhibitors in plants subjected to drought stress. The reviewed transgenic studies are mainly on the model plant Arabidopsis. The study of plant gene function using gain-of-function or loss-of-function mutants, such as overexpression or knockout/down, has been reviewed (Kondou et al., 2010; Bolle et al., 2011). However, the pros and cons of the above-mentioned experimental validation systems are outside the scope of the current review and, thus, will not be discussed. Readers are directed to excellent reviews where such issues are critically discussed (Bajaj et al., 1999; Sharma et al., 2002; Bhatnagar-Mathur et al., 2008; Kondou et al., 2010; Bolle et al., 2011; Rabilloud and Lescuyer, 2014; Rhee and Mutwil, 2014; Khan et al., 2016; Handler et al., 2018).

**Table 4:** Studies showing differential functional activities of proteases in plants under drought stress.

Plant species	Tissue	Genotypes	Functional assay	Differential functional activities of proteases under drought stress	References
**Wheat**	Leaves	Katya-resistant Pobeda-susceptible Sadovo-susceptible	Endopeptidase and aminopeptidase activity assays	Increased endopeptidase activity in all three wheat cultivars. The susceptible varieties exhibited the highest activity.	Simova-Stoilova et al. (2010)
**Common bean**	Leaves	Tiber-tolerant Starozagorski-susceptible Zorin-susceptible	Endoproteolytic and aminopeptidase activity assays	Serine protease activity highest in drought-susceptible varieties. Increased activity of aminopeptidase in Starozagorski.	Hieng et al. (2004)
**Cowpea &** **Common bean**	Leaves	EPACE-1-tolerant TI83D- tolerant Carioca-sensitive IPA-sensitive	Endoproteolytic activity assays	Increased endoproteolytic activity higher in drought-susceptible bean cultivars compared to drought tolerant cowpea cultivars. The highest activity of aspartic protease was observed in Carioca, the most drought-susceptible bean cultivars.	Cruz de Carvalho et al. (2001)

**Table 5:** List of transgenic overexpression studies illustrating the roles of protease and protease inhibitors in plants under drought stress.

Gene ID	Name	Transgenic plant	Drought-induced performance of transgenic plants	References
Proteases				
*ASPG1*	*Aspartic protease in guard cell 1*	Arabidopsis	Overexpression of *ASPG1* gene in drought-stressed Arabidopsis plants enhanced ABA sensitivity in guard cells and triggered stomatal closure to reduced water loss. The transgenic plants also showed increased activities of superoxide dismutase and catalase compared to wild-type plants. The *ASPG1* gene played an essential role in drought avoidance through ABA signalling in guard cells and displayed more tolerance.	Yao et al. (2012)
*APA1*	*Aspartic Protease*	Arabidopsis	*APA1* expression was induced in response to drought stress and ABA treatment in the transgenic plants. The overexpression of the *APA1* gene in the transgenic plants enhanced drought tolerance to mild water deficit through ABA-dependent signalling.	Sebastián et al. (2020)
*SPCP2*	*Sweet potato papain-like cysteine protease*	Arabidopsis	Transgenic Arabidopsis plants overexpressing *SPCP2* exhibited early flowering, higher percentage of incompletely developed siliques, reduced average fresh weight and lower seed germination rates compared to the wild-type. *SPCP2* gene expression was induced during natural leaf senescence in salt and drought stress tolerance compared to wild-type plants.	Chen et al. (2010)
Protease Inhibitors				
*BBI*	*Bowman-Birk inhibitor*	Arabidopsis	The transgenic plants expressing *BBI* maintained normal growth and higher relative water content when compared to the wild-type plants. The transgenic plant overexpressing *BBI* showed increased activities of glutathione-s-transferase and ascorbate peroxidase antioxidants and reduced malondialdehyde content when compared to the wild-type plants. The expression of *BBI* in the transgenic plants enhanced drought tolerance.	Malefo et al. (2020)
*OCPI1*	*Oryza sativa chymotrypsin inhibitor-like 1*	Rice	*OCPI1* overexpressing transgenic plants had higher total protein content than wild-type under drought stress. *OCPI1* overexpressing plants also had higher grain yield compared to the wild-type. Chymotrypsin activity was also inhibited in the *OCPII*-overexpressing transgenic plant.	Huang et al. (2007)
*OCP12*	*Oryza sativa chymotrypsin inhibitor-like 2*	Arabidopsis	*OCPI2* overexpressing plants maintained higher biomass, relative water and proline content compared to the wild-type. Chymotrypsin protease activities were lower in the transgenic compared to the wild-type plants. The transgenic plants overexpressing *OCP12* showed enhanced tolerance to salt and osmotic stresses when compared to wild-type plants.	Tiwari et al. (2015)
*MpCYS4*	*Malus prunifolia* cystanin	Arabidopsis and Apple	Transgenic plants overexpressing *MpCYS4* exhibited higher relative water content, chlorophyll content and stomatal closure, and reduced electrolyte leakage compared to the wild-type. Overexpression of MpCYS4 in transgenic plants led to up-regulation of ABA-and drought-related genes and enhanced drought tolerance.	Tan et al. (2017)

ABA-abscisic acid.

**Table 6:** List of transgenic knockout/down studies illustrating the roles of protease and protease inhibitors in plants under drought stress.

Gene ID	Name	Transgenic plant	Drought-induced performance of knockout plants	References
*ZmSAG3*	*Zea mays Senescence-associated gene*	Maize	Increased levels of MDA, ion leakage, ROS content, wilting and senescence in *ZmSAG3-OE* compared to wild-type. Increased seed germination rate and levels of antioxidant enzymes, high levels of chlorophyll synthesis and stress-related genes in knockout plants with reduced levels of senescence and chlorophyll degradation-related genes compared to wild-type.	Wang et al. (2022)
*HvPap1* & *HvPap19*	*Cathepsin F-like & Cathepsin B-like genes*	Barley	*HvPap1* knockdown plants exhibited delayed leaf senescence under control conditions, thicker cuticle under control and drought conditions. Jasmonic acid and JA-isoleucine levels increased in *HvPap1* knockdown and wild-type plants following drought. ABA content high in wild-type, and the two knockdown lines, was significantly higher in knockdown plants after drought.	Gomez-sanchez et al. (2019)
*Tr-KPI*	*Kunitz proteinase inhibitor*	White clover	High proline levels before and after drought stress in *tr-kdi1* and *tr-kdi5* knockdown plants than wild-type. Increased drought-induced expression of genes involved in ethylene biosynthesis in knockdown plants.	Islam et al. (2017)
*AtFtsHi3*	*Arabidopsis thaliana Pseudo-protease AtFtsHi3*	Arabidopsis	*ftshi3-I(kd)* knockdown lines had a pale green phenotype, reduced growth, and distorted chloroplast ultrastructure following drought stress but high endogenous ABA content. Early onset of drought sensitivity in wild-type and *ftshi3-1*(*Comp)* lines that *ftshi3-I(kd)*. Increased levels of ABA-responsive genes in the *AtFTsHi3* knockdown mutant than the wild-type plants.	Mishra et al. (2021)

ABA, abscisic acid; MDA, malondialdehdye; ROS, reactive oxygen species; kd, knockdown.

##### 2.2.2.1 Enzyme activity assays

Using a wide range of substrates and protease inhibitors, Hieng et al. (2004) evaluated the activities of different proteolytic enzymes in leaf extracts of common bean (*Phaseolus vulgaris*) cultivars subjected to water limitation. The cultivars used, Tiber, Zorin and Starozagorski exhibited various degrees of sensitivity to drought stress, with Tiber being more drought-tolerant. The results showed a general decrease in leaf protein content in all three cultivars, possibly due to increased proteolytic activity. However, the drought-tolerant cultivar exhibited the least protein content decrease and the least amount of proteolytic activity under severe drought stress. Activity assays for a 65 kDa serine protease with a pH optimum of 8.5 showed the greatest increase in the drought-susceptible varieties Starozagorski and Zorin, and a decrease in Tiber, the tolerant cultivar under severe stress. In contrast, the activities of two other serine proteases increased in all three cultivars, possibly illustrating the existence of a large group of proteases with different roles under drought stress in common beans. The study also reported increased activity of an aminopeptidase in the most drought-sensitive Starozagorski and a decrease in the drought-tolerant cultivar. Overall, the findings of the study support the notion of the presence of a wide range of proteolytic enzymes with different substrate specificities, subcellular locations and specific inhibitors in plants. The authors also suggested that regulation of serine proteases in the tolerant variety could guard against premature drought-included leaf senescence in common bean (Hieng et al., 2004).

In another study, Simova-Stoilova et al. (2010) investigated the activities of acidic proteases and aminopeptidases in winter wheat genotypes under drought and recovery at biochemical and transcriptional levels. The cultivars used were Katya (drought-resistant), Pobeda (cold-resistant) and Sadovo (disease-resistant), with Pobeda and Sadovo being more drought-sensitive than Katya. The results showed significant variations in leaf total protein content of control plants of all three cultivars. Furthermore, upon exposure to water limitation, the protein content of the drought-susceptible Pobeda and Sadovo varieties decreased by almost 50% relative to the respective controls but was restored after re-watering. On the other hand, minimal protein loss was observed in the drought-resistant cultivar under drought stress. The results also showed increased endopeptidase activity for all three cultivars under severe drought stress, with both susceptible varieties exhibiting the highest activity. The high basal level of azocaseinolytic activity observed in the drought-resistant variety, but the least drought-induced effect is noteworthy. As suggested in sorghum transcriptomics studies under water limitation (Fracasso et al., 2016; Azzouz-Olden et al., 2020) high constitutive expression of genes in drought-tolerant genotypes could contribute towards superior traits to dehydration stress. Overall, the authors suggested that low levels of proteolytic activity observed in the drought-resistant Katya cultivar could be regarded as a marker for drought tolerance (Simova-Stoilova et al., 2010).

Likewise, Cruz de Carvalho et al. (2001) investigated the leaf endoprotease activity of common bean and cowpea (*Vigna unguiculata)* cultivars in response to drought stress. The cowpea cultivars EPACE-1 and TI83D were reportedly more drought-tolerant than the common bean cultivars Carioca and IPA, with an overall tolerance trend of EPACE-1 > TI83D > IPA > Carioca. The results showed differences in endoproteolytic activity across all four cultivars, being higher in the most drought-susceptible common bean cultivars than in cowpea. For example, under mild-drought conditions, the total endoproteolytic activity increased by 235%, 119%, 95% and 58% for cultivar Carioca, IPA, TI83D and EPACE-1, respectively. The study also investigated the involvement of cysteine, aspartic, serine, and metalloproteases in the drought response of the most drought-sensitive Carioca cultivar using class-specific protease inhibitors. The results suggested limited involvement of metalloproteases in the common bean cultivar under mild stress, but some enzyme activity of cysteine and serine proteases. Furthermore, pepstatin A inhibited about 25% of the total proteolytic activity under mild-drought conditions, thus indicating the presence of drought-responsive aspartic proteases in the bean leaf extract. Subsequent analysis of aspartic protease activity in all four cultivars using a specific peptide substrate revealed that water deficit induced aspartic protease activity in cowpea and bean leaf tissues. However, this enzyme activity increased with increasing levels of sensitivity to drought. The authors suggested that this increase in aspartic protease enzyme activity in the drought-sensitive bean cultivars could be a drought-adaptive response for remobilizing nitrogen to other plant parts under stressful environments (Cruz de Carvalho et al., 2001).

##### 2.2.2.2 Transgenic plant biology

###### 2.2.2.2.1 Using overexpression mutant lines

Drought studies using transgenic plants overexpressing protease and protease inhibitors genes (**Table 5**) are unravelling the roles of protein degradation under conditions of water deprivation (Huang et al., 2007; Zhang et al., 2008; Chen et al., 2010; Yao et al., 2012; Tiwari et al., 2015; Tan et al., 2017; Roux et al., 2019; Malefo et al., 2020; Sebastián et al., 2020). For example, Yao et al. (2012) conducted an extensive molecular study of an Arabidopsis gene *ASPG1* (*aspartic protease in guard cell* 1) and its biological functions in drought response. The study used wild-type and *ASPG1-*overexpressing *(ASPG1-*OE) Arabidopsis plants to investigate tissue-specific gene expression patterns and the roles of *ASPG1* in ABA-signalling systems associated with drought. After exposure to a 14-day drought stress treatment followed by re-watering, *ASPG1-*OE plants exhibited a greater recovery from wilting symptoms and higher survival rates. Gene expression analysis revealed that *ASPG1* is drought-inducible in both wild-type and *ASPG1-*OE plants and is expressed in young seedlings, leaves, stems, flowers, and siliques but not in roots. The gene is also preferentially localized in guard cells where it participates in the ABA-signalling processes, including ROS accumulation triggering stomatal closure. The increased hydrogen peroxide contents are further detoxified by high antioxidant activities of superoxide dismutase and catalase, thus alleviating oxidative stress effects. In addition, increased ABA sensitivity was observed in *ASPG1-*OE plants under drought stress which could account for increased stomatal closure and a significant reduction in transpiration water loss. The authors suggested that *ASPG1* participates in drought response *via* the ABA-dependent pathway (Yao et al., 2012).

Sebastián et al. (2020) also investigated the participation of an aspartic protease gene (*APA1*) in drought response using transgenic Arabidopsis plants overexpressing *APA1* (OE-*APA1*), wild-type and *apa1* insertional lines under well-watered and mild-water deficit conditions. The study revealed that OE*-APA1* lines tolerated drought stress better than the wild-type and *apa1* lines. In addition, drought stress did not affect the overall plant phenotype, total chlorophyll content, and principal root length of the OE*-APA1* lines relative to the well-watered plants. However, *APA1* overexpressing plants exhibited reduced stomatal pore aperture and water loss under mild-drought stress compared to the wild-type and *apa1* lines, yet exogenous ABA did not intensify stomatal closure. *APA1* was shown to be ABA-responsive, exhibiting a 4-fold increase in expression in well-watered wild-type plants supplemented with exogenous ABA. Furthermore, under well-watered conditions, OE-*APA1* plants up-regulated the expression of ABA biosynthetic and signalling genes relative to the wild-type, while the same genes were down-regulation in *apa1* insertional lines. As such, the authors suggested that *APA1* participates in drought response *via* the regulation of the ABA-signalling pathway and its location in leaves, vascular tissues, epidermal, and guard cells implicate it in stomatal closure as a water-saving mechanism under conditions of water scarcity (Sebastián et al., 2020).

Likewise, Chen et al. (2010) also investigated the role of sweet potato (*Ipomoea batatas*) papain-like cysteine protease (*SPCP2)* in transgenic Arabidopsis plants. Qualitative phenotypic analyses showed that transgenic Arabidopsis plants overexpressing *SPCP2* contained a higher number of incompletely developed siliques, and exhibited earlier flowering, reduced fresh weight per seed, and lower germination rates compared to the wild-type controls. It is plausible that the SPCP2 protein degraded silique storage proteins resulting in incomplete development. Furthermore, *SPCP2* gene expression was induced during natural leaf senescence, thus suggesting a gene function in senescence. *SPCP2* transgenic Arabidopsis plants also showed high tolerance to salt and drought stresses compared to the wild-type, further implicating the gene in osmotic-stress response (Chen et al., 2010).

Bowman-Birk inhibitors (BBI) are compound inhibitors that inhibit both trypsin and chymotrypsin (Habib and Fazili, 2007; Vaseva et al., 2012). Malefo et al. (2020) generated transgenic *A. thaliana* plants overexpressing a *BBI* gene from maize and studied its role in drought tolerance. Drought stress was imposed on 4-week-old plants by withholding water for 9 days, and comparisons were made between the wild-type and transgenic plants under well-watered and drought-stress conditions. The results showed that the transgenic plants exhibited less wilting symptoms, greater survival rate, higher RWC, and higher fresh leaf biomass than the wild-type under drought conditions. Furthermore, lipid peroxidation was reduced, while glutathione-S-transferase activity was enhanced in transgenic lines compared to the wild-type exposed to water limitation. The authors suggested that the improved performance of the *BBI*-overexpressing transgenic plants under drought could be attributed partly to the reduction in drought-induced oxidative stress (Malefo et al., 2020).

In another study, an *Oryza sativa chymotrypsin inhibitor-like 1* (*OCPI1*) gene was transformed into rice plants and characterized at the reproductive stage under drought-stressed field conditions (Huang et al., 2007). The results showed that the positive transgenic plants inhibited endogenous chymotrypsin activity resulting in higher total protein content of leaves and panicles compared to the wild-type under drought conditions. Furthermore, the *OCPI1*-overexpressing plants had higher grain yield due to higher seed setting rates than the wild-type under similar drought conditions, further supporting the potential usefulness of *OCPI1* in crop improvement strategies (Huang et al., 2007). Likewise, the over-expression of an *OCPI2* gene in Arabidopsis resulted in enhanced tolerance to salt and PEG- and mannitol-induced osmotic stresses in transgenic plants relative to the wild-type (Tiwari et al., 2015). Transgenic Arabidopsis *OCPI2* plants also exhibited greater vegetative and reproductive potential, higher biomass, seed yield, RWC, membrane stability and proline content. The authors suggested that these enhanced characteristics could have contributed towards the better performance of transgenic plants under salt and osmotic stresses (Tiwari et al., 2015).

Apart from serine protease inhibitors discussed above, plants also contain cystatins which inhibit cysteine proteases of papain and legumain families (Grudkowska and Zagdańska, 2004; Martínez et al., 2012). Some phytocystatins are drought-inducible as evidenced by increased gene expression of a triticale cystatin *TrcC-8* in triticate leaf and root tissue under dehydration (Chojnacka et al., 2015). Western blotting analysis also showed increased drought-induced accumulation of the protein but its decline upon re-watering in the same tissues. In addition, the recombinant TrcC-8 protein exhibited inhibitory effects on wheat and triticale leaf cysteine proteases under water deficit stress (Chojnacka et al., 2015). Therefore, cystatins may function as regulatory elements of cysteine protease activity under drought stress (Diop et al., 2004; Zhang et al., 2008).

Tan et al. (2017) studied the biological roles of cystatins under drought stress by overexpressing a *Malus prunifolia* cystatin gene (*MpCYS4*) in transgenic Arabidopsis and apple (*Malus domestica*). The study showed that the *MpCYS4* protein is localized in the nucleus, plasma membrane and cytoplasm, consistent with its known cellular locations (Kidric et al., 2014; Diaz-Mendoza et al., 2016). A range of drought assays showed that transgenic Arabidopsis plants overexpressing *MpCYS4* had improved drought tolerance as exhibited by the plants’ decreased water loss, increased survival rate, increased RWC and greater stomatal closure under drought, relative to the wild-type. Physiological assessments of the transgenic apple plants overexpressing *MpCYS4* also exhibited higher RWC, chlorophyll content and stomatal closure, and reduced electrolyte leakage under water deficits compared to the wild-type. Generally, the *MpCYS4* gene resulted in higher transcriptional expression of known ABA- and stress-responsive genes in both transgenic Arabidopsis and apple plants, further highlighting the involvement of *MpCYS4* in ABA-signalling processes and thus drought tolerance (Tan et al., 2017).

###### 2.2.2.2.2 Using knockout or knockdown mutant lines

Plant gene function can also be studied using knockout or knockdown mutants with total or partial loss of gene function (Bolle et al., 2011). Several studies have utilized such mutants of proteases or protease inhibitors to study their role during plant growth and drought response (Islam et al., 2017; Gomez-sanchez et al., 2019; Mishra et al., 2021; Wang et al., 2022). A summary of these studies is also given in **Table 6**. For example, Wang et al. (2022) investigated the role of the maize (*Z. mays*) senescence-associated gene, *ZmSAG39* encoding a cysteine protease in maize plants subjected to either darkness or drought stress. The genotypes used were wild-type, transgenic *ZmSAG39* overexpression (OE) lines and *ZmSAG39* knockout lines. The results indicated that *ZmSAG39* gene expression was induced by darkness and drought stress in the leaves of wild-type plants. The study also investigated seed germination rates in wild-type, *ZmSAG39* knockout and *ZmSAG39-*OE plants under 8% and 12% polyethylene glycol (PEG)-6000 treatment. The results showed that the knockout lines had higher germination rates when compared to the wild and *ZmSAG39*-OE. Furthermore, the *ZmSAG39-*OE lines exhibited severe leaf senescence under complete darkness for 5 days, with lower chlorophyll content but higher membrane ion leakage rate and malondialdehyde (MDA) content than wild-type and knockout lines.

Similarly, following a 14-day drought-stress treatment imposed by withholding water, the *ZmSAG39-*OE lines showed greater levels of leaf wilting and senescence than the wild-type. In contrast, the *ZmSAG39* knockout lines had an upright and greener phenotype. In addition, the *ZmSAG39-*OE lines exhibited reduced survival rates and chlorophyll content, higher membrane leakage levels, MDA, ROS, and oxidative stress damage compared to wild-type and knockout lines. Interestingly, the activities of a range of antioxidant enzymes were much greater in the *ZmSAG39* knockout lines than in the wild-type and *ZmSAG39-*OE lines, thus, indicating the enhanced antioxidant capacity of knockout mutants. The expression levels of various stress-responsive genes were also analyzed between the wild-type and transgenic plants. The results showed expression levels of stress-related genes (lipid-transfer protein, caleosin-related family protein, and salt-overly sensitive 1) and chlorophyll synthesis-related genes (chlorophyll *a* oxygenase) were higher in the drought-stressed knockout lines compared to the wild-type.

On the other hand, senescence-related genes (microRNA 3, cysteine protease, and senescence-associated gene 12) and a chlorophyll degradation-related gene (non-yellow colouring 1) were higher in the drought-stressed *ZmSAG39-*OE lines compared to the wild-type. These results indicate the negative regulatory effect of *ZmSAG39* on the analysed stress-related and chlorophyll-synthesis-related genes under drought stress. However, under the same conditions of water deficits, *ZmSAG39* exhibited a positive regulatory effect on genes associated with senescence and chlorophyll degradation. The authors concluded that *ZmSAG39* is associated with leaf senescence, and its increased expression under darkness and drought enhances the sensitivity of maize plants to stress. As such, the knocking out of *ZmSAG3* from maize enhanced the resistance of maize plants to darkness and drought tolerance and delayed leaf senescence (Wang et al., 2022).

Gomez-sanchez et al. (2019) also investigated the effect of individually knocking down drought-induced cysteine protease genes, *HvPap1 and HvPap19*, in leaves of barley (*Hordeum vulgare*) under drought stress. Seven-day-old plants of wild-type, knockdown *HvPap1* (*KDPap1*), and knockdown *HvPap19* (*KDPap19*) were subjected to drought stress by withholding water for 14 days. The wild-type plants were more susceptible to water deprivation, as evidenced by reduced leaf turgor, while both knockdown genotypes remained upright. Furthermore, *KDPap1* plants exhibited delayed leaf senescence during their normal growth cycle under well-watered conditions compared to the wild-type. *KDPap1* also had a thicker upper epidermis cuticle under normal and drought conditions compared to the wild-type and *KDPap19*. However, the drought-induced reduction in chlorophyll and carotenoid contents was not significantly different between the three genotypes. Nevertheless, drought-stressed *KDPap1* and *KDPap19* plants exhibited higher total protein content than wild-type plants, possibly indicating reduced proteolysis in the knockdown mutant lines.

The study also analysed the levels of selected phytohormones in the three genotypes under well-watered and drought-stress conditions (Gomez-sanchez et al., 2019). The results showed differences in the responses of the phytohormones to water deprivation. For example, ABA levels increased upon exposure to drought in wild-type, *KDPap1* and *KDPap19* lines, possibly underscoring the critical role of this hormone during drought response. However, ABA levels were much higher in both knockdown lines compared to the wild-type. Conversely, 12-oxo-phytodienoic acid (OPDA), a precursor for jasmonic acid (Taiz and Zeiger, 2012), decreased in all three genotypes following water deprivation. This decline could be attributed to the observed increase in jasmonic acid and its bioactive form, jasmonic-isoleucine, in drought-stressed wild-type and *KDPap1* plants. However, independent lines of *KDPap19* gave inconsistent trends in jasmonic acid and jasmonic-isoleucine levels. On the other hand, salicylic acid remained unchanged under drought stress conditions in wild-type and transgenic plants. Although increases in ABA and jasmonic acid levels are known to regulate stomatal closure and water loss under drought stress conditions (Salvi et al., 2021), the observed stomatal behaviour in the knockdown plants was unusual. The authors, however, concluded that *HvPap1 and HvPap19* are drought-inducible genes and may cause differential effects on barley plants subjected to drought (Gomez-sanchez et al., 2019).

Islam et al. (2017) investigated the functions of Kunitz proteinase inhibitors (KPIs) in white clover (*Trifolium repens* L.) plants subjected to water deficits using several knockdown lines of different *Tr-KPIs*. The study utilised two distinct water withholding regimes, a Non-PreStress treatment with direct exposure of plants to water deficits and a PreStress treatment in which plants were initially exposed to drought stress and rehydration, followed by a final drought stress treatment. Both *Tr-KPI1* and *Tr-KPI5* genes were drought-inducible in three untransformed white clover genotypes with varying degrees of drought tolerance. However, significantly higher gene expression levels were observed in the PreStress treatment, and the genes exhibited different induction levels in leaf and root tissues. The study also showed that proline accumulation and gene expression of *9-cis-epoxycarotenoid dioxygenase* (*NCED1*), a key enzyme in the ABA biosynthesis pathway (Taiz and Zeiger, 2012) are drought-inducible in Non-PreStress treated plants but not PreStress treatment. The authors suggested that PreStress water deprivation treatment primes plants for future exposure to water limitation, resulting in reduced proline accumulation and no significant changes in *NCED* gene expression.

To further study the role of *Tr-KPI* genes in drought response, four knockdown mutant lines were generated, *35S::tr-kpi1*, *35S::tr-kpi2*, *35S::tr-kpi4* and *35S::tr-kpi5*; however, for the sake of brevity in this review, the knockdown lines will be referred to as *kpi1*, *kpi2*, *kpi4* and *kpi5*, respectively. Leaf proline levels were much higher in well-watered plants of *kpi1* and *kpi5* lines and even greater during PreStress treatment than the wild-type plants. However, *kpi2* and *kpi4* lines exhibited lower proline levels under well-watered conditions compared to the wild-type and were not used further in the study. These results possibly highlight the additive effects of high endogenous proline levels in *kpi1* and *kpi5* lines in response to PreStress treatment. Both *kpi1* and *kpi5* plants also exhibited increased drought-induced transcript abundance of ethylene biosynthesis genes, 1-aminocyclopropane-1-carboxylic acid (ACC) synthase 1 (ACS1) and ACC oxidase 1 (ACO1) compared to the wild-type plants. The authors suggested that knockdown lines may experience some level of constitutive stress under well-watered conditions; hence the increased proline accumulation possibly acts as a ROS scavenger. Furthermore, *Tr-KPIs* may have different tissue-specific expression patterns and target various proteases, resulting in a functionally diverse group of active proteinase inhibitors during plant growth and drought response (Islam et al., 2017).

In an extensive study, Mishra et al. (2021) investigated the effect of an *A. thaliana filamentous temperature-sensitive H* (*FtsH*) pseudo-protease (*AtFtsHi3*) on the growth and drought tolerance of Arabidopsis plants using knockdown mutants. Pseudo-proteases are catalytically inactive proteolytic enzymes with cell regulatory functions (Reynolds and Fischer, 2015). Gene expression analysis of *AtFtsHi3* indicated that the transcript was expressed in seedlings, young flowers, roots, leaves, siliques, and stems during the growth of wild-type Arabidopsis plants. The generated knockdown mutant, *ftshi3-I (kd)*, exhibited a 99% reduction in the transcript expression of the target gene *AtFtsHi3* relative to the wild-type. In addition, the mutant seedlings and plants had a pale-green phenotype, were much smaller in rosette size, and had reduced root growth, with lower numbers of lateral roots than the wild-type. The study also generated complementation lines by expressing the *FtsHi3* gene in the *ftshi3-I (kd)* background and designated them *ftshi3-I (Comp)*. Further analysis of chloroplasts ultrastructure between the wild-type, *ftshi3-I (Comp)*, and *ftshi3-I (kd*) showed that the knockdown mutant lines had distorted chloroplasts with thinner membranes, fewer and distorted thylakoid membranes, and fewer starch granules relative to the other wild-type and *Comp* lines.

Severe drought stress was also imposed on 4-week-old Arabidopsis plants of wild-type, *ftshi3-I (Comp)*, and *ftshi3-I (kd*) lines by withholding watering for up to 20 days and observing the drought-related phenotypes. The results showed that *ftshi3-I (kd*) plants were more tolerant to 12-days of drought treatment, while the wild-type and *ftshi3-I (Comp)* plants were more drought sensitive with wilting and chlorotic phenotypes. Furthermore, 80% of the *ftshi3-I(kd*) plants recovered after 20 days of drought stress, followed by 14 days of re-watering, while only 20% of the wild-type and *ftshi3-I (Comp)* recovered under the same conditions. While all three genotypes had relatively similar leaf ABA levels under control conditions, upon exposure to water limitation, the wild-type and *ftshi3-I (Comp)* plants had higher ABA content in leaves than the knockdown line. The authors suggested that high endogenous levels of ABA in cotyledons and roots under well-watered conditions could better prime the *ftshi3-I(kd*) plants for drought than the wild-type. Furthermore, the *ftshi3-I(kd*) plants had reduced stomatal density but larger stomatal size than the other two genotypes. Overall, this study showed that *ftshi3-I(kd*) plants had improved drought tolerance compared to wild-type and *ftshi3-I (Comp)* plants (Mishra et al., 2021).

### 2.3 Overall functions: more than just acts of protein degradation and its regulation

**Figure 2:**
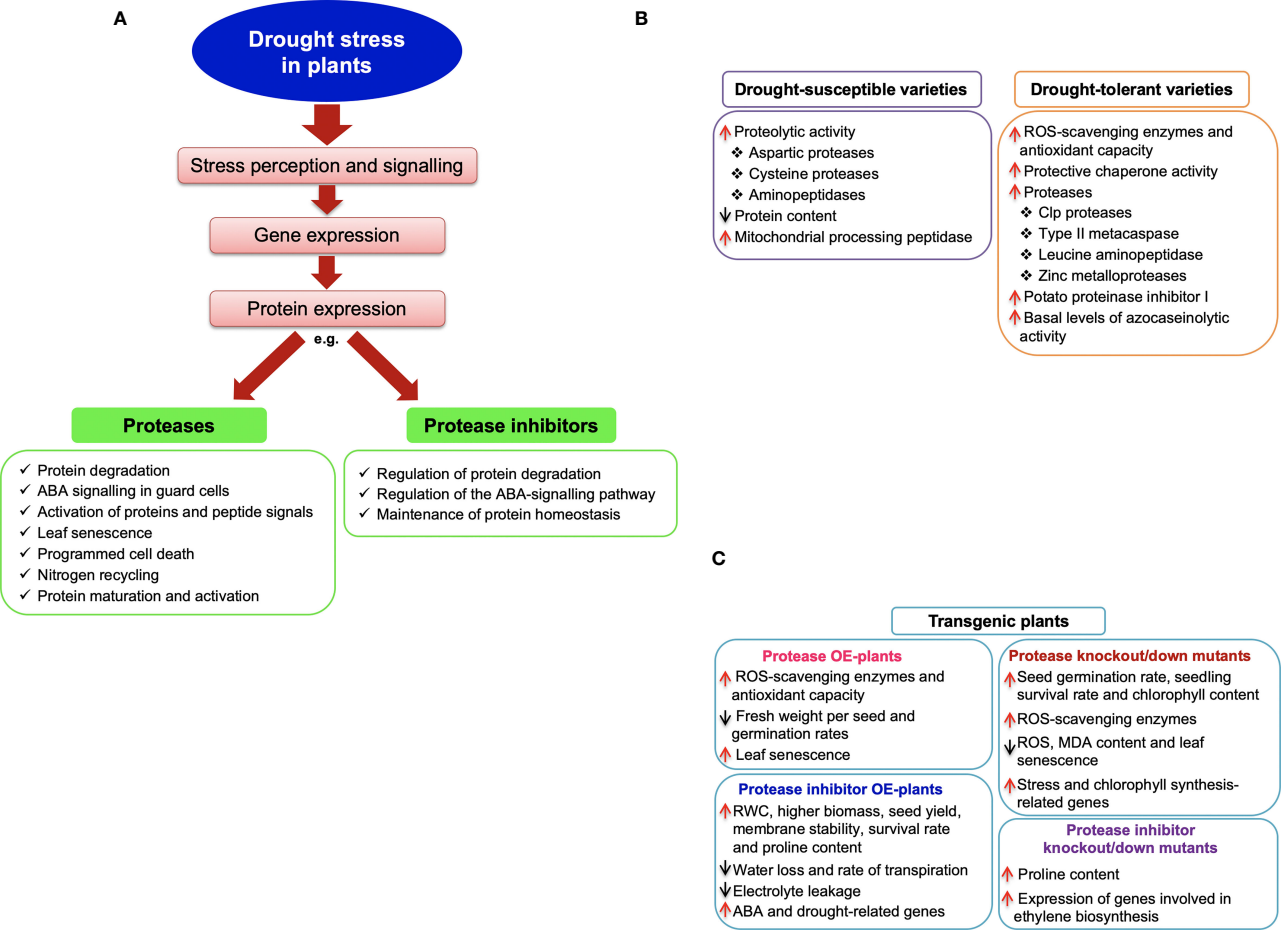
Regulation and functions of plant proteases and protease inhibitors extrapolated from various drought-stress studies. **(A)** A simplified schematic diagram showing that drought stress signalling in plants results in changes in gene and protein expression patterns such as those of proteases and protease inhibitors. **(B)** Differential regulation of plant proteases and protease inhibitors in drought-susceptible and tolerant plant varieties under drought stress. **(C)** Physiological effects of transgenic overexpression and repression of proteases and protease inhibitors in plants under drought stress. Red arrows indicate up-regulation or increase, while black arrows indicate down-regulation or decrease. ABA, abscisic acid; Clp, caseinolytic protease; MDA, malondialdehyde; ROS, reactive oxygen species; RWC, relative water content; OE-plants, overexpression-plants. (Cruz de Carvalho et al., 2001; Hieng et al., 2004; Huang et al., 2007; Chen et al., 2010; Simova-Stoilova et al., 2010; Ford et al., 2011; Yao et al., 2012; Ashoub et al., 2013; Jedmowski et al., 2014; Faghani et al., 2015; Tiwari et al., 2015; Wang et al., 2015; Cheng et al., 2016; Chmielewska et al., 2016; Wu et al., 2016; Islam et al., 2017; Tan et al., 2017; Gomez-sanchez et al., 2019; Goche et al., 2020; Malefo et al., 2020; Sebastián et al., 2020; Mishra et al., 2021; Wang et al., 2022).

In summary, plants encounter drought-induced osmotic and oxidative stresses, which disrupt cell structure and function (Levitt, 1980b; Ingram and Bartels, 1996; Bray, 1997). In turn, drought signalling events alter the expression patterns of various genes and proteins with diverse functions in drought adaptation, such as proteases and protease inhibitors (**Figure 2A**). Consequently, damaged, misfolded, or aggregated proteins are either removed through protein degradation activities of proteases (Vierstra, 1996; Vaseva et al., 2012) or stabilized by chaperons and osmoprotectants (Ingram and Bartels, 1996; Bray, 1997) to restore cellular homeostasis. Such proteolytic activities are essential for preventing the accumulation of potentially toxic non-functional proteins and peptides, recycling nitrogen sources and providing free amino acid pools for renewed protein synthesis well-suited for stressful conditions (Vierstra, 1996; van Wijk, 2015; Rowland et al., 2022). The accumulated drought-responsive protease inhibitor proteins primarily regulate the catalytic activities of proteases (Mosolov and Valueva, 2011). Nonetheless, the roles of plant proteases and their inhibitors under drought stress are more than just acts of protein degradation and regulation (**Figure 2**).

Results from comparative proteomics and enzyme activity assays (**Figure 2B**) and transgenic biology studies (**Figure 2C**) provide experimental evidence for the diverse roles of plant proteases and their inhibitors under drought stress (**Tables 3**-**6**). For example, while most plants undergo protein damage due to osmotic and/or oxidative stresses, the drought-sensitive genotypes suffer the most, as evidenced by their increased endoproteolytic activities and reduced protein content under drought stress (Cruz de Carvalho et al., 2001; Hieng et al., 2004; Simova-Stoilova et al., 2010). On the contrary, drought-tolerant genotypes protect their proteins from drought-induced damage by increasing the efficiency of their protective machinery, such as antioxidant capacity and chaperon activities, and inhibiting proteolysis (Cheng et al., 2016; Goche et al., 2020). Furthermore, the reviewed transgenic plant biology studies (**Tables 5**, **6**) add new insights into other roles of proteases and protease inhibitors, including maintaining high RWC and water conservation through enhanced stomatal control (Yao et al., 2012; Tiwari et al., 2015; Tan et al., 2017; Malefo et al., 2020; Sebastián et al., 2020). In addition, proteases and their inhibitors participate in ABA-signalling events and the up-regulation of ABA-responsive genes essential for drought adaptation (Yao et al., 2012; Tan et al., 2017; Sebastián et al., 2020). As a result, transgenic plants overexpressing either protease or protease inhibitor genes exhibit enhanced performance, plant growth and yield, survival rate, and resilience to water deficits (Huang et al., 2007; Yao et al., 2012; Tiwari et al., 2015; Tan et al., 2017; Malefo et al., 2020).

Furthermore, gene function studies using loss-of-function technologies such as knockout/down mutants also provide additional experimental evidence on the broader roles of plant proteases and their inhibitors in drought response (**Tables 5**, **6**; **Figure 2C**). Nevertheless, the experimental challenges related to the unintended effects of gene manipulation technologies on the overall plant phenotype are real. For example, Gomez-sanchez et al. (2019) suggested that the observed unexpected increase in cuticle thickness of *KDPap1*, an *HvPap1* knockdown mutant line, could have been due to pleiotropic effects associated with the knocked-down cysteine protease gene. In addition, knockdown mutants exhibit some residual mRNA expression or protein accumulation of the knocked-down gene to varying extents (Islam et al., 2017; Gomez-sanchez et al., 2019; Mishra et al., 2021), hence the knockdown character (Gomez-sanchez et al., 2019). For example, while Mishra et al. (2021) reported a 99% reduction in the transcript expression of the target *AtFtsHi3* gene in the generated knockdown mutant, *ftshi3-I (kd)*, relative to the wild-type, other studies reported much lower reduction rates of the target genes (Islam et al., 2017).

Furthermore, although the knockdown procedure would have specifically intended to reduce the expression level of a target gene, in some cases, untargeted genes are also affected (Islam et al., 2017). For example, Islam et al. (2017) produced different knockdown mutants of specific *Tr-KPIs* in their study. However, the RNA interference procedure also affected the expression of other untargeted members of the *Tr-KPI* gene family. Consequently, evaluating resultant plant mutant phenotypes after exposure to drought stress becomes challenging. Therefore, despite the steady progress in gene function investigations using transgenic mutant plants, more studies with multiple transgenic lines are required to ascertain the *bona fide* physiological effects and roles of target protease and protease inhibitor genes.

As discussed earlier, phytohormones may interact during plant growth and development, as well as in response to drought stress, ultimately contributing towards the overall plant phenotype (Clarke and Durley, 1981; Hale and Orcutt, 1987; Ullah et al., 2018; Jogawat et al., 2021; Salvi et al., 2021; Iqbal et al., 2022). In some cases, the proteolytic machinery regulates phytohormone activity and signalling (Ullah et al., 2018; Salvi et al., 2021; Iqbal et al., 2022). This is evidenced by the results of the reviewed transgenic studies (**Tables 5**, **6**) involving genes of aspartic proteases (Yao et al., 2012; Sebastián et al., 2020), a cystatin (Tan et al., 2017), senescence-associated gene (Wang et al., 2022), cathepsin-like proteases (Gomez-sanchez et al., 2019), Kunitz proteinase inhibitors (Islam et al., 2017) and a pseudo-protease FtsHi3 (Mishra et al., 2021).

For example, gibberellins regulate gene expression through DELLA proteins, which are repressors of plant growth and development (Achard and Genschik, 2009). Under normal growth conditions, gibberellins facilitate optimal plant growth and development by mediating the 26S proteasome degradation of DELLA proteins. Conversely, when exposed to drought conditions, gibberellins levels decrease, and DELLA proteins accumulate, resulting in plant growth inhibition and the development of dwarf plant phenotypes (Achard and Genschik, 2009; Taiz and Zeiger, 2012; Colebrook et al., 2014; Salvi et al., 2021). Similarly, the 26S proteasome degradation pathway is involved in the jasmonic acid signalling pathway (Taiz and Zeiger, 2012). During drought stress, the biosynthesis and accumulation of jasmonic acid increases and jasmonate ZIM-domain (JAZ) proteins are degraded by the 26S proteasome, thus allowing transcription of downstream stress-related genes. JAZ proteins are repressors of DNA-binding transcriptional factors of the jasmonate hormonal signalling system (Taiz and Zeiger, 2012; Wager and Browse, 2012). Therefore, their presence under normal growth conditions regulates the transcriptional control of jasmonic acid-related gene expression (Wager and Browse, 2012). Thus, the 26S proteasome machinery regulates phytohormone signalling systems in plants.

**Figure 3:**
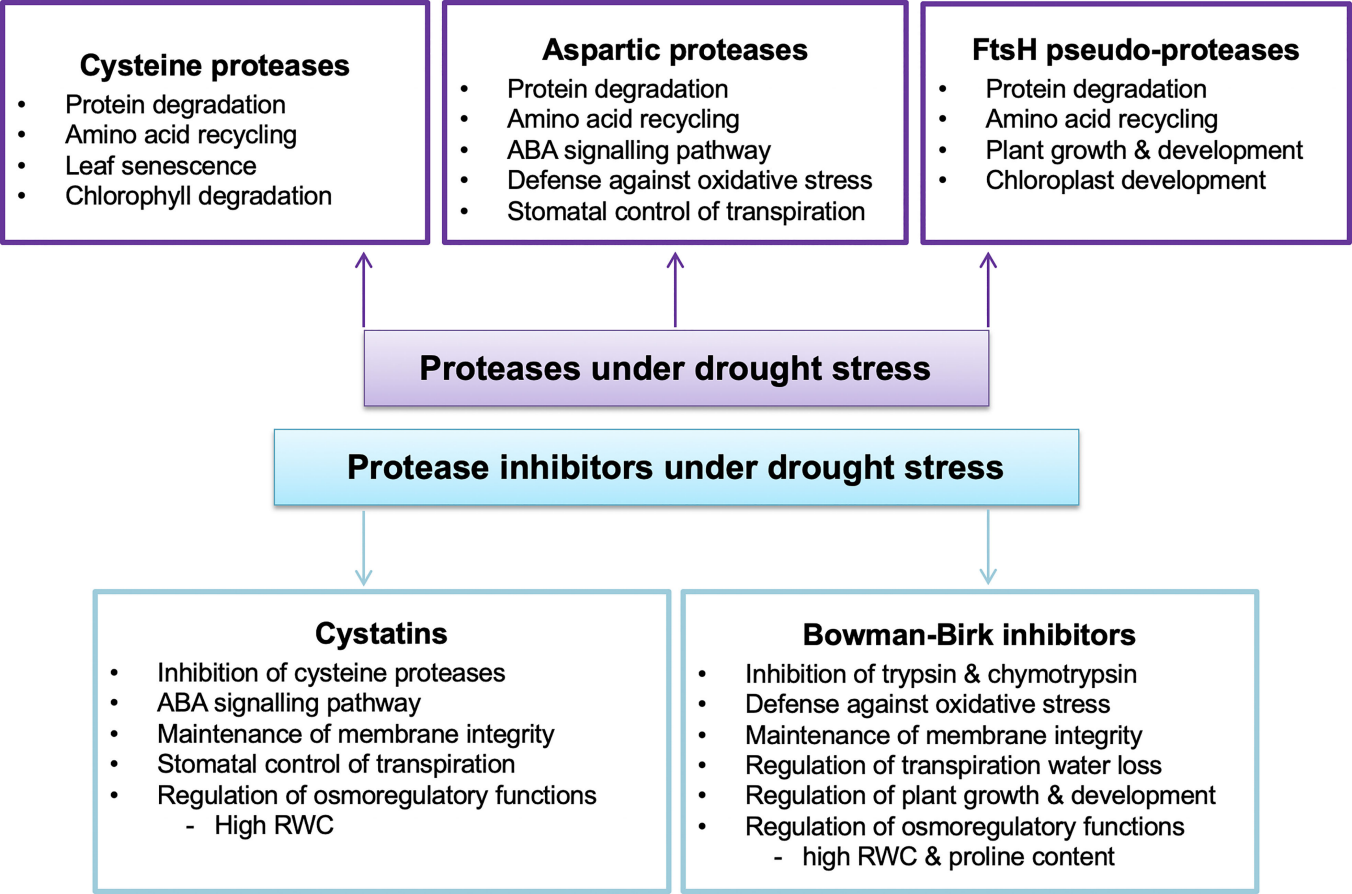
Physiological functions of selected proteases and protease inhibitors in drought-stressed plants inferred from transgenic studies. ABA, Abscisic acid; RWC, relative water content. (Chen et al., 2010; Yao et al., 2012; Tan et al., 2017; Malefo et al., 2020; Sebastián et al., 2020; Mishra et al., 2021).

Other studies have reported that exogenous application of ABA increases chymotrypsin inhibitory activity in barley leaves, while jasmonic acid treatment enhances trypsin inhibitor activity (Casaretto et al., 2004). These observations highlight the role of phytohormones in regulating proteolytic activities and vice versa during normal plant growth and drought response. Indeed, the current review of comparative proteomics, enzyme activity assays and transgenic studies expands our understanding of the broader functions of proteases and protease inhibitors in drought response. Some of the inferred physiological roles of protein degradation activities and their regulatory processes discussed above are summarised in **Figure 3**.

## 3 Concluding remarks and perspectives

Protein degradation and its regulatory processes in plants are crucial for maintaining cellular homeostasis under water deficits; otherwise, damaged proteins would accumulate and disrupt cell structure and metabolism. However, the drought-induced differential expression of proteases and protease inhibitor proteins in contrasting genotypes are also unravelling the complex networking events during drought responses in plants. For example, increased proteolysis and reduced total protein in drought-sensitive genotypes could indicate massive protein damage events and less robust protective machinery against drought and its secondary effects, which result in sub-optimal metabolic and growth processes. In contrast, the more drought-tolerant genotypes exhibit reduced levels of proteolysis due to fewer proteases and more protease inhibitors. Together with enhanced protective machinery and improved targeted stress-induced gene transcriptional and translational activities, these factors collectively contribute towards greater performance and survival rates under water-limitation conditions.

Nevertheless, the types and functions of proteases and protease inhibitors are quite diverse, and these proteins could be involved in many more physiological roles, such as stomatal control and ABA, jasmonic acid, and ethylene signalling. Furthermore, with this wide structural and functional diversity, their differential expression patterns between drought-tolerant and susceptible genotypes possibly make proteases and protease inhibitors important groups of proteins with potential use in crop improvement strategies. For instance, could the reduced level of protein degradation in drought-tolerant genotypes be used as a marker for drought tolerance and vice versa? Nevertheless, more functional validation studies are required to unravel additional roles of protein degradation events in plants under drought stress as we continue to find ways of generating crops with enhanced resilience to hot and drier climates. Furthermore, meta-analyses of protease and protease inhibitor gene and/or protein expression datasets between contrasting plant species and genotypes are required to unravel the expansive role of protein degradation in plant drought response.

## Author contributions

Conceptualisation, RN. Writing-manuscript preparation, SJM, RN. Writing-review and editing, RN. Funding acquisition, supervision, RN. Both authors contributed to the article and approved the submitted version.
